# A Mixed Methods Case Study of Food Shopping in a Community with High Infant Mortality

**DOI:** 10.3390/nu13113845

**Published:** 2021-10-28

**Authors:** Sarah Evenosky, Eleanor Lewis, Katherine I. DiSantis

**Affiliations:** 1 College of Health Sciences, Arcadia University, Glenside, PA 19038, USA; 2 Sidney Kimmel Medical College, Thomas Jefferson University, Philadelphia, PA 19107, USA; Eleanor.Kane@students.jefferson.edu; 3 College of Population Health, Thomas Jefferson University, Philadelphia, PA 19107, USA; Katie.DiSantis@jefferson.edu

**Keywords:** pregnancy, nutrition, food environment, birth equity, health disparities, underserved populations

## Abstract

In the U.S., preterm birth disproportionately impacts certain racial/ethnic groups, with Black women experiencing preterm birth at a rate 50% higher than other groups. Among the numerous factors that likely contribute to these increased rates are neighborhood characteristics, such as food environment. In this mixed-methods case study, we evaluated how pregnant women living in a predominately minority, lower income community with high preterm birth rates navigate and perceive their food environment. Qualitative interviews were performed to assess perceptions of food environment (*n* = 7) along with geographic and observational assessments of their food environment. Participants traveled an average of 2.10 miles (SD = 1.16) and shopped at an average of 3 stores. They emphasized the importance of pricing and convenience when considering where to shop and asserted that they sought out healthier foods they thought would enhance their pregnancy health. Observational assessments of stores’ nutrition environment showed that stores with lower nutritional scores were in neighborhoods with greater poverty and a higher percent Black population. Future policies and programmatic efforts should focus on improving nutrition during pregnancy for women living in communities with high rates of poor birth outcomes. Availability, affordability, and accessibility are key aspects of the food environment to consider when attempting to achieve birth equity.

## 1. Introduction

### 1.1. Birth Inequities and Race

Preterm birth, defined as spontaneous or provider-initiated birth before 37 weeks’ gestation, remains the leading cause of death among children under five years old and is associated with short- and long-term neonatal morbidity in the U.S. [1,2,3]. Black women are particularly at risk for delivering early, experiencing a preterm birth at rate 50% higher than all other racial/ethnic groups in the U.S. [4]. Among factors found to play a role in preterm birth are neighborhood racial composition and the level of neighborhood deprivation. Greater proportions of residents from predominantly Black or more deprived neighborhoods (i.e., neighborhoods that have median household incomes below the national level) were associated with low birthweight and preterm birth [5,6,7]. The mechanisms for increased preterm birth among Black women and/or women living in deprived neighborhoods is not clearly established, but a likely contributor is access to nutritious foods [8,9].

### 1.2. Neighborhood Characteristics and Dietary Quality

Numerous systematic factors, including segregation and biased urban planning practices, creates a poor nutritional environment for residents of low-income and Black communities, making it more difficult for women to follow the established nutrient standards and dietary intake guidelines recommended during pregnancy [10,11,12]. Neighborhoods with larger proportions of Black residents and/or greater poverty often have limited access to healthy foods through fewer high-quality food stores, higher prices for food, and fewer food options [13,14,15]. A recent study found that Black residents of a “food swamp” (an area with high access to low-quality food) have a lower diet quality than Black residents with better food environments [16]. Another study with women of lower income and reproductive age found that residents of census tracts with a higher proportion of Black residents, had lower individual level consumption of fruits and vegetables [17].

### 1.3. Pregnancy Health Outcomes and Food Environment

The health of a pregnancy is impacted by the affordability, accessibility, and availability of healthy foods within a women’s community [11,18]. For example, pregnant women who live further than 1.5 miles from a supermarket are three times more likely to have intrauterine growth restriction (IUGR), where the fetus is born with a low birth weight due to restrictions in growth during pregnancy [14]. Similarly, higher numbers of convenience stores within a one-mile radius of a pregnant woman’s household have been associated with a higher risk of preterm birth and low birth weight [19]. Further research has linked food insecurity (i.e., inconsistent access to adequate and nutritious food) and poor diet quality to pregnancy morbidity, including gestational diabetes, IUGR, and preterm birth [15,20,21].

Diet quality, including low consumption of nutrient dense foods (e.g., vegetables, fruits, dairy) and high consumption of foods with added sugar and fast foods, has been associated with preterm birth and low birthweight [22,23]. Poor diet quality can also lead to micronutrient deficiencies, including iron, folate, and iodine deficiencies, which have been linked to adverse pregnancy outcomes, such as miscarriage and preterm birth [11,24,25,26].

### 1.4. Current Study

Given the evidence, we used a mixed methods approach to evaluate how pregnant women living in a predominantly Black/Latino community with high preterm birth rates navigate their food environment. Use of a mixed methods approach in this study allowed for a more complete picture of experiences [27], combining geospatial data on income and race at the neighborhood level as well as in depth perspectives from qualitative interviews of those living in these neighborhoods. Understanding the experience of food shopping for women living in communities with high rates of preterm birth holds potential to offer insights into intervention strategies that would improve healthy food access during pregnancy. On a more global level, Black and Hispanic women in Norristown represent an understudied group that experiences high rates of infant mortality. This small city environment is representative of many small- to moderate-size cities which might lack the transportation and other infrastructure resources that larger cities have for their residents [28]. Therefore, this study aims to highlight barriers and potential areas of intervention, in an underserved and minority community [29].

## 2. Materials and Methods

### 2.1. Setting

Norristown, a small city in southeastern Pennsylvania, was the setting of this study. Norristown had a preterm birth rate of 11%, between 2014 and 2016, which was higher than the national average of 9.6% at the time [30,31]. The city has a population of approximately 34,400 residents, moderate median household income of $49,895 (compared to the national and statewide median household income of $67,871 and $69,960, respectively), and racial diversity (34.36% non-Hispanic black, 32.99% non-Hispanic white, and 25.62% Hispanic) [32]. The small city is within Montgomery county, which is 75% White and is the second wealthiest county in the state. Within this community, black mothers experience preterm delivery at a rate of 13.5%, while white mothers experience preterm birth at 8.4% [31]. A study on birth outcomes in Montgomery County found that Norristown has the highest number of Black infant and fetal deaths of all cities in the county [33]. We recruited from a center that provides social services, including access to a food pantry and free prenatal health classes, and serves predominately lower income and Black and Hispanic women that live within the surrounding neighborhood.

### 2.2. Sample Recruitment

Participants were recruited at pregnancy support groups offered by parent educators at a non-profit social service agency within Norristown. To participate, women had to be Norristown residents and be currently pregnant or have given birth within the previous 12 months, and English-speaking. Recruitment involved passive and active methods, including posting flyers at the social service agency and in-person at the beginning or end of the scheduled support group meetings. Before beginning any study activities, all participating women gave written or verbal informed consent. Arcadia University’s Institutional Review Board approved this research.

### 2.3. Procedures

Participants completed semi-structured interviews using a moderator guide, followed by a short demographic/descriptive survey. One interview was completed over the phone for convenience of the participant, while the rest were completed in person. The moderator guide focused on:

1. Food shopping patterns over the previous four weeks (e.g., frequency, transportation);
2. Identification of any factors that impact where they choose to shop (e.g., price, closeness of store, healthiness, safety of store location, family preferences, etc.);
3. Challenges/frustrations related to their food shopping experiences;
4. The impact of pregnancy on food shopping and/or dietary behaviors.

The moderator guide included all open-ended questions with optional probing questions to help the participants provide further detail in their answers. Regarding factors that impact where they shop, closed-ended questions, followed by “why,” were used if the participant did not specifically mention pricing, closeness, healthiness, or safety of the store, to ensure that all variables were addressed.

For mapping purposes, the participants were asked for the closest intersection to their home address as an approximate location for their residence. Survey data were collected to describe the sample and to support the interview transcripts, which included demographic variables, nutrition supplementation program participation and pregnancy history. All interviews were audio recorded and transcribed. Participants received a baby gift of $25 value following completion of the research activities.

#### Neighborhood Food Environment Assessment

All grocery stores that participants reported they shopped at within the last four weeks were evaluated using the Nutrition Environment Measures Survey in Stores (NEMS-S). The NEMS-S focuses on the availability and pricing of healthier foods and is a reliable and validated tool for measuring a store’s nutrition environment [13]. Grocery stores were defined as those stores having three or more registers and a fresh produce section offering at least six varieties. Per the NEMS-S protocol, 11 categories of food products (milk, fruit, vegetables, ground beef, hot dog, frozen dinners, baked goods, beverages, bread, chips, and cereal) were assessed for availability and pricing; fresh fruits and vegetables were also assessed for quality. The total summary score for each grocery store was calculated by adding the total points earned in each of the three measures (availability, quality, and price), for a total of up to 54 points max. Scores were used to provide more context around the shopping patterns of the participants.

### 2.4. Data Analysis

#### 2.4.1. Qualitative Analysis

Interviews were transcribed verbatim and coded using NVivo software. Conventional content analysis was used to identify themes that helped explain participants’ shopping patterns and to describe perceptions of the food environment in general, and relative to pregnancy experience [34].

#### 2.4.2. Geospatial Analysis

Individual participant maps were created in ArcGIS Online, an online free software that is part of the Esri geospatial cloud. Maps included a geospatial distribution of racial and income level data throughout Norristown, as obtained from different publicly accessible ArcGIS map layers created using census level data [35]. The approximate location of the participant’s house and all the food stores shopped at within the previous four weeks were included on the maps. Maps allowed for a visual representation of the food environment of each woman participating in the study and the travel patterns of their food shopping behaviors. Distance traveled, in miles, was calculated from the participant’s respective home location to each of the food stores they reported shopping at using the direction function on Google maps. A descriptive analysis was performed in SPSS Software to describe the distance traveled by an individual participant to grocery stores and to present the stores’ respective NEMS-S scores.

## 3. Results

### 3.1. Participant Characteristics

All women (*n* = 7) reported they lived in Norristown. Five participants were pregnant at the time of the interview and two participants had already given birth approximately one and two months prior to the interview. Participant demographics are outlined in Table 1. All participants were pregnant with or gave birth to a singleton, and birth order ranged from first-born to eighth-born. Pregnancy timing, gravidity, and parity for the women are described in Table 1. Two participants reported a history of preterm deliveries, one at 35 weeks and the other at 36 weeks and 6 days. All women reported they began receiving prenatal care in the first trimester of pregnancy.

### 3.2. Description of Shopping Patterns

On average, participants reported traveling to three stores in the previous four weeks to buy food for themselves and their families. Of the eight total stores reported by participants, five fit the criteria of a grocery store. The other three could be categorized as dollar stores, drug stores, and discount department stores. Each participant reported shopping at one grocery store at a minimum. Overall, participants traveled an average distance of 2.10 miles (SD = 1.16) when shopping for food, with the largest mean distance being 3.3 miles (SD = 2.55). Three participants reported traveling further than four miles to a food store in the previous four weeks. Of the women interviewed, five out of seven reported having a car they used to go food shopping, while two reported walking and sometimes taking a cab to go food shopping.

### 3.3. Qualitative Themes

On average, interviews lasted 13 min (SD = 3.44), with an average transcript length of five and a half pages. Three themes were identified:

1. Monetary considerations and barriers;
2. Convenient, but still good quality;
3. Thing I buy and do not buy because I’m pregnant.

The first two helped explain the shopping patterns of women living in the city, while the third focused on the relationship between nutrition and pregnancy health.

#### 3.3.1. Monetary Considerations and Barriers

As explained by the participants, monetary factors were paramount in deciding where to shop and when to buy particular items. Several participants expressed the importance of prices, sales, and deals available to them when choosing where to shop. For example, one participant explained why she buys microwaveable meals from the dollar store instead of from a corner/convenience store: “that means that I’m spending an extra $1.50 on something that I know if I went to the dollar store and got the same thing, I’m saving a whole $1.50.” Some participants also explained they would travel farther if they knew food was going to be cheaper.

This theme was commonly described in the context of budgeting and making do with the amount of money and/or food stamps participants have to spend/use. A common challenge was “not having enough money for it, running out of money quickly.” Those participants who received federal food assistance from SNAP and/or WIC described the difficulty of ensuring that their SNAP dollars (also called “food stamps” by participants) lasted them the whole month. For example, one participant explained her experience of why she shops for the lowest price:

“Because we’re on a budget. We are a one job, right now, income family with three of us so…we do get food stamps, so we’re limited to what we can buy and how much we can spend, and I need to make sure that I make that…last throughout the month.”

#### 3.3.2. Convenient, but Still Good Quality

Participants stressed the importance of convenience when food shopping. This theme prevailed across participants regarding the proximity of the store location and ease of food shopping amongst other daily tasks. As described by one participant who reported walking to food stores, “that’s like the closest store, so you can’t really be choosey.” The participants who stated they drove to food stores reported planning food shopping trips in conjunction with other daily tasks. For instance, one participant explained, “today will be a day when I’m out. So, after I leave here, I’ll go to [Store A] because it’s right around the corner…get my waters and stuff and then take care of whatever else I got to take care of one way around.” Participants cited the ability to buy more than just food at or near a store as a motivating factor for choosing a where to shop.

While paying attention to convenience, women still reported they would like to be receiving a certain level of food quality. However, the women sampled commonly expressed difficulties finding quality foods in the grocery stores. According to one participant, “some of the meats…look like…old or like they’re about to expire,” and “their produce…it doesn’t look good, like it seems like it’s been sitting out for too long, like nobody’s buying it.”

When considering the intersection between convenience and quality, women reported choosing a closer store over a further one due to pregnancy-related fatigue. One participant explained shopping less often since becoming pregnant: “I haven’t really been shopping that much because I’ve been, it’s the first trimester, so I’ve been kind of tired.” Prioritizing convenience over other factors was frequently explained in relation to the pregnancy experience and was especially common among women who had many other people at home.

#### 3.3.3. Things I Buy and Do Not Buy Because I Am Pregnant

Participants discussed buying healthier food and maintaining healthier diets during pregnancy. Foods that were frequently identified as healthier and reportedly eaten more frequently during pregnancy included fruits, vegetables, salads, meat, and milk. Participants also reported increasing their water intake to stay hydrated, as recommended by their providers. Additionally, participants described eating less “snacks,” “junk,” and “fatty” foods, such as tasty cakes, marshmallows, fried foods, chips, ice cream, soda, and caffeine. Two participants described how their diagnoses of diabetes (gestational and type II) impacted their decisions to limit sugary food and drink consumption during pregnancy.

Participants related efforts to purchase “healthier” foods during pregnancy to their diet’s impact on the health of their baby. As one participant stated: “I’m more conscious of reading what’s in the foods before I buy them because it’s not just my body that I’m destroying possibly now.” Another participant stated, “what I eat is going straight to the baby. What I’m nourishing myself with is nourishing the baby.” Many participants also identified the importance of weight gain during pregnancy, since they were eating for two people.

### 3.4. Geospatial Analysis

**Figure 1:**
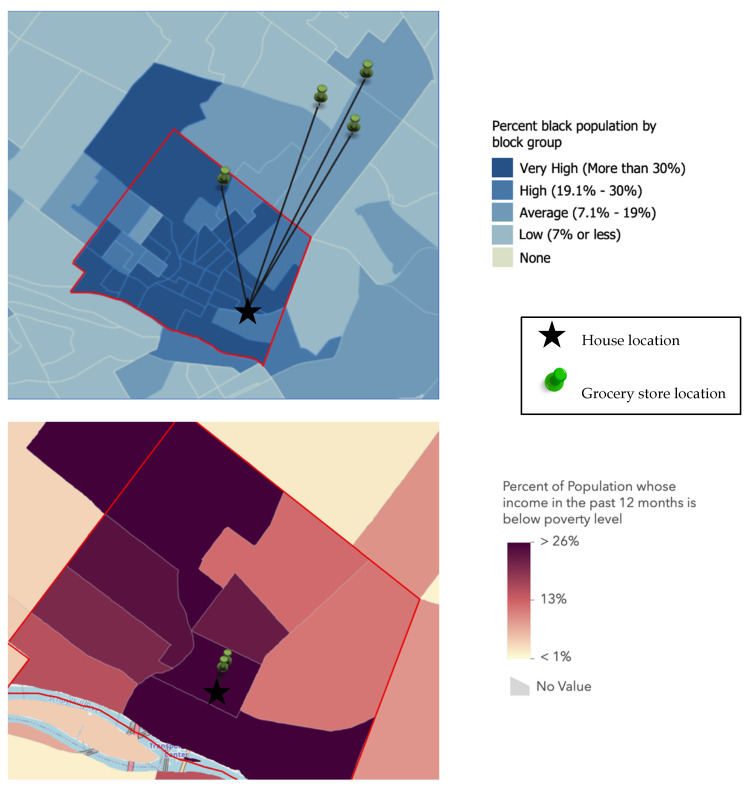
Maps for two participants, showing their home location and all the stores they reported shopping at in the previous four weeks.

Figure 1 provides a visual geographic representation of two participants’ shopping patterns in relation to their home location and either neighborhood racial distribution or poverty level. The participants frequently traveled outside of the city to go food shopping. Only two participants, those who reported primarily walking to food stores, exclusively shopped within the boundaries of the city.

### 3.5. Quantitative Assessment of Food Environment

The five grocery stores that the participants utilized were assessed with the NEMS-S. The NEMS-S scores ranged from 22 to 40 out of 54, where stores within city limits received the lowest scores (22 and 24) relative to stores in neighboring suburbs. When looking at all 14 reported shopping trips (ranging from 1–3 trips per participant), stores within 1 mile of home (*n* = 3) had a mean NEMS-S score of 23. Compared to stores more than 1 mile from the home (*n* = 11), which had a mean NEMS-S score of 33. NEMS-S results are presented in Table 2, in relation to neighborhood poverty level and racial distribution (data obtained from ArcGIS online). The data demonstrate that grocery stores with lower NEMS-S scores were in neighborhoods with a higher prevalence of poverty and that had a predominantly Black population.

## 4. Discussion

This study aimed to understand women’s food shopping experiences during pregnancy in a racially diverse and low socioeconomic status city with high rates of preterm births and infant mortality, at the community level. Women living in this city described pricing (i.e., fitting within budget), convenience, and quality of foods offered as being paramount when deciding where to shop. They also described a preference for choosing a closer store due to pregnancy-related fatigue or transportation obstacles, yet closer stores did not always offer healthier, high quality foods in their experience. Mapping analyses showed patterns consistent with previous research, demonstrating that grocery stores with higher quality products and better prices were in the non-minority and higher-income communities, surrounding this predominately minority, lower income city [12,36]. The limited access to healthy grocery stores has worrisome implications for healthy eating in pregnancy, and for reducing disparities in U.S. birth outcomes.

One strategy to address limited access to healthy foods has been to add new full supermarkets to neighborhoods without them. Yet this strategy has shown mixed results in terms of its ability to increase healthful food purchasing among residents [37,38]. The availability of many less healthy items at supermarkets likely contributes to the mixed impact on overall food purchasing and consumption [37,38]. Other important aspects raised by the women have been assessed through in-store interventions. A recent systematic review found that interventions using in-store promotions (e.g., shelf-tags, sampling products) can improve the healthfulness of food purchases [39]. Another systematic review examined nutrition interventions in food stores within low-income urban neighborhoods and found point-of-purchase signage and product placement strategies (e.g., endcap displays) were the most effective in promoting healthier food purchases [40]. In an area with low access to supermarkets, embracing models that can work with stores that are more plentiful and therefore proximal to more residents, such as convenience stores and small grocers, holds promise. Thus, widespread efforts to bring in-store nutrition interventions to small stores in lower income neighborhoods could enhance awareness of healthy products nearby and perhaps encourage retailers to increase their offerings of healthier items [41].

Some strengths and limitations of this study should be considered. The mixed methods approach allowed for a deep understanding of the experiences of pregnant women living and shopping in a community with high rates of pre-term birth. While the sample size was small, the methods provided as in-depth assessment based on objective and perceived measures of food environment, along with maps of travel patterns for food shopping. It should be acknowledged that the lack of data on dietary intake or foods purchased do not allow for conclusions about whether shopping patterns impacted consumption patterns in these women. While the results provided details on how pregnant women in this community navigate their food environment, the study design does not allow for direct correlations to be drawn between the food availability, healthfulness of diets, and the pregnancy outcome of preterm birth.

## 5. Conclusions and Future Research

These findings support further inquiry into the impact of neighborhood food environments on improving pregnancy outcomes. They highlight the importance of considering the food environment when attempting to reduce preterm birth at the population level. In this small city with a relatively high rate of preterm birth, women valued the potential role of healthier foods in managing their health during pregnancy yet had to travel further for healthy food options. Future policies and programmatic efforts to reduce birth inequities should include improvements to existing food environments and/or mitigation of current barriers to healthy food access in the environment, particularly those predicated by community development that does not favor predominately lower income and/or Black neighborhoods. Proximity, convenience, store preferences, food pricing and food preferences should all be considered when attempting to achieve birth equity by way of food environments.

## Figures and Tables

**Table 1:** Demographics and characteristics of participants.

**Demographics**	***n***
Age (years)	
20–29	2
30–40	4
>40	1
Race & Ethnicity	
Non-Hispanic black	2
Non-Hispanic white	3
Non-Hispanic Black/white mixed	2
Highest Level of Education	
Some high school	2
High school graduate/GED	2
Technical school or associates degree	1
Some college	2
Household Income ^1^	
<$20,000	5
$35,000–$49,999	1
Federal Food Assistance ^2^	
SNAP	6
WIC	3
**Pregnancy Characteristics**	***n***
Timing	
1st trimester	2
2nd trimester	1
3rd trimester	2
Postpartum	2
Gravidity	
1	3
≥2	4
Parity	
0	2
Primiparous	1
Multiparous	4

^1^ One participant selected “not willing to share” for this survey question. ^2^ SNAP = Supplemental Nutrition Assistance Program; WIC = Special Supplemental for Womens, Infant, and Children; Families can be eligible for both SNAP and WIC in the State of Pennsylvania.

**Table 2:** NEMS-S Score of Grocery Stores Categorized by Neighborhood Type.

Neighborhood Type		NEMS-S Score
% Poverty	% Black Population	Total Summary Score
33.7	38.6	22
28.2	34.0	24
9.1	14.6	34
6.1	8.0	37
2.6	10.2	40

Note. NEMS-S = Nutrition Environment Measures Survey in Stores. Neighborhood type was categorized by poverty level (by census tracts) and racial distribution (by block group) of the neighborhood in which the store is located. Poverty level was measured as the percent of the population whose income was below the poverty level, in the past 12 months. Racial distribution data were provided as the percent of black population within each block group.

## Data Availability

Maps throughout this article were created using ArcGIS^®^ software by Esri. ArcGIS^®^ and ArcMap™ are the intellectual property of Esri and are used herein under license. Copyright © Esri. All rights reserved. For more information about Esri^®^ software, please visit www.esri.com, accessed on 15 August 2021.
